# Evaluation of a Custom Three‐Dimensional Surgical Guide in Mandibular Sagittal Split Osteotomy: An Exploratory Split‐Mouth Randomized Controlled Trial

**DOI:** 10.1111/ocr.70163

**Published:** 2026-07-16

**Authors:** Evy Eida Vitria, Revini Nuita, Muhammad Kamil Hassan, Nissia Ananda, Tan Wui Jing, Zakiah Mat Ripen, Norliza Ibrahim

**Affiliations:** ^1^ Department of Oral and Maxillofacial Surgery, Faculty of Dentistry Universitas Indonesia DKI Jakarta Jakarta Pusat Indonesia; ^2^ Department Oral and Maxillofacial Clinical Sciences, Faculty of Dentistry Universiti Malaya Kuala Lumpur Malaysia; ^3^ Dental Unit, Universitas Indonesia Hospital Universitas Indonesia Depok West Java Indonesia

**Keywords:** inferior alveolar nerve, orthognathic surgery, sagittal split ramus osteotomy, three‐dimensional printing

## Abstract

**Objective:**

This exploratory split‐mouth randomized controlled trial aimed to evaluate the effect of a custom three‐dimensional (3D) surgical guide on mandibular sagittal split osteotomy outcomes compared with conventional freehand osteotomy.

**Methods:**

Patients undergoing sagittal split osteotomy (SSO) were randomly assigned in a split‐mouth design to receive mandibular osteotomy performed with a custom‐made 3D surgical guide on one side and without a surgical guide on the contralateral side. The primary outcome was the postoperative lingual split scale (LSS) of the mandible. Secondary outcomes were lateral osteotomy end (LOE), assessed using postoperative CT‐based evaluation, and intraoperative inferior alveolar nerve exposure (NE).

**Results:**

Fifteen patients contributed 30 mandibular sides for paired analysis. Guided sides showed more favourable LSS (*p* = 0.041) and LOE (*p* = 0.041) distributions compared with non‐guided sides. No statistically significant difference was observed in NE (*p* = 0.763). These findings suggest that the use of a custom 3D surgical guide may improve osteotomy control and split predictability.

**Conclusion:**

The custom 3D surgical guide was associated with more favourable lingual split pattern and lateral osteotomy end distributions during sagittal split osteotomy. Given that the study population consisted predominantly of skeletal Class III patients, these findings should be interpreted primarily within the context of Class III patients. No significant reduction in intraoperative inferior alveolar nerve exposure was observed.

## 1 Introduction

Sagittal split osteotomy (SSO) is one of the most commonly performed orthognathic surgeries, with or without upper jaw surgery. This procedure is versatile and can be used for correcting dentofacial deformities including mandibular prognathism, retrognathism, asymmetry, open bite, and skeletal malocclusions [1, 2]. Hugo Obwegeser introduced the bilateral sagittal split osteotomy in 1957 [2]. This technique resulted in a significant increase in the buccal plate of the proximal osteotomy segment as it included a much larger proportion of the mandibular ramus area. The concept was later refined by Dal Pont in 1959, who suggested increasing the buccal surface area by extending osteotomy into the proximal mandibular body area [3]. The Hunsuck–Epker modification differs from the Obwegeser technique in the extent of the lingual osteotomy [4]. Nowadays, the Obwegeser–Dal Pont–Hunsuck and Epker modification are frequently used to advance or set back the mandible [5].

Despite being a well‐established procedure, sagittal split osteotomy remains a challenge for the surgeon on account of the technical difficulty. One of the main challenges is the lack of direct visual control of the lingual split scale that occurs during the split procedure [6]. A bad split in SSO is defined as an unfavourable fracture pattern of the mandible during osteotomy [7]. Bad splits are known to increase the risk of postoperative complications, including infection, temporomandibular joint problems, and fibrous rather than bony healing [8]. Key anatomical variations, including lingula height, mandibular ramus thickness, and proximity of the inferior alveolar canal to the buccal cortex, have been shown to influence osteotomy outcomes [9, 10]. The reported incidence of bad split ranges from 0.5% to 5.5% [10], with a large‐scale study by Jiang et al. reporting an incidence of 4.15% [11].

The transition from conventional two‐dimensional (2D) planning to three‐dimensional (3D) virtual surgical planning has rapidly advanced in orthognathic surgery [12]. Custom‐made 3D surgical guides have emerged as a promising method to improve osteotomy accuracy and minimize intraoperative complications [13]. Using 3D models, the lingual surface of the mandible can be visualized in detail, enabling observation of the previously hidden lingual aspect of the fracture line [14]. These guides translate virtual osteotomy planning into intraoperative execution with high fidelity, potentially increasing accuracy, minimizing human error, and reducing mechanical torsion forces during splitting [15].

Previous studies have reported that dental occlusal–surface supported cutting guides can assist bone cutting and drilling during SSO, providing accurate positioning and improved surgical precision in mandibular osteotomy procedures [16]. In addition, an in vitro proof‐of‐concept study demonstrated that 3D‐printed cutting and drilling guides for guided SSO achieved high positional accuracy, with deviations of less than 0.17 mm from the virtual surgical plan, supporting the role of patient‐specific guides in enhancing osteotomy precision [17]. Although the use of mandibular cutting guides for SSO has been reported in previous studies, limited studies have evaluated the use of custom 3D surgical guides in SSO using a split‐mouth randomized design, particularly in relation to lingual split scale, lateral osteotomy end, and inferior alveolar nerve exposure. The aim of this study was to evaluate and compare the postoperative lingual split scale (LSS) of the mandible, the lateral osteotomy end (LOE) at the inferior border, and position of the inferior alveolar nerve following osteotomy, between conventional and guided methods.

## 2 Materials & Methods

This study was designed as an exploratory, assessor‐blinded, interventional split‐mouth randomized controlled trial. Each participant served as their own control: one mandibular side underwent SSO with a custom 3D surgical guide (intervention) and the contralateral side underwent conventional freehand SSO (control). The allocation ratio was 1:1 at the mandibular side level. Eligible participants underwent SSO, with or without concomitant maxillary surgery, at Universiti Malaya Medical Centre and University of Indonesia Hospital from February 2022 to December 2025. Inclusion criteria were age ≥ 18 years, mandibular skeletal Class II or Class III deformity, and written informed consent. Exclusion criteria were non‐SSO orthognathic procedures, medically compromised patients, and craniofacial syndromic patients. The overall study workflow, participant enrollment, randomization, and allocation are summarized in Figure S1.

Randomization of the mandibular side was performed using computer‐generated simple randomization. Allocation concealment was achieved using sealed, opaque envelopes prepared by an independent investigator. The allocation was opened intraoperatively immediately prior to osteotomy. This approach was intended to reduce selection bias and improve allocation concealment. Blinding of surgeons was not feasible; however, postoperative CT datasets were anonymized and coded so that outcome assessors and data analysts remained blinded. There were no major changes made to the trial design, eligibility criteria, interventions, or primary and secondary outcomes after trial commencement. All outcome measures and analyses were prespecified in the study protocol. There were no losses to follow‐up or protocol deviations, so the total sample analysed matched those who were enrolled. The trial was stopped after the enrolled samples met the minimum sample size. All enrolled participants completed the study and were included in the final analysis. All patients received standard perioperative care, and no differential concomitant treatment was administered between intervention and control side. Inter‐rater reliability was used to assess agreement between two independent raters (first and sixth author) interpreting postoperative CT scans.

Ethical approval was obtained from the Faculty of Dentistry Ethics Committee, Universiti Malaya (DF OS2211/0024 [P]); Faculty of Dentistry, University of Indonesia (78/Ethical Approval/FKGUI/V/2025); and the Ethics Committee of the University of Indonesia Hospital (S‐159/KETLIT/RSUI/VII/2025). The trial was registered in the International Randomized Controlled Trial Number (ISRCTN) with trial number ISRCTN35139147 and reported according to CONSORT guidelines [18]. The study was carried out without any deviations from the original protocol, and there were no procedural changes after the trial began.

### 2.1 Guide Design, Digital Workflow, and Fabrication

A single‐piece cutting guide was designed to transfer the virtual osteotomy plan intraoperatively (Figure 1). The guide incorporated a posterior ‘lingula stop’ to define the endpoint of the medial horizontal osteotomy just posterior to the lingula within the fossa region (Figure 1B). It is inserted from the anterior ramus and secured with one or two self‐drilling screws for stability during osteotomy.

**FIGURE 1:**
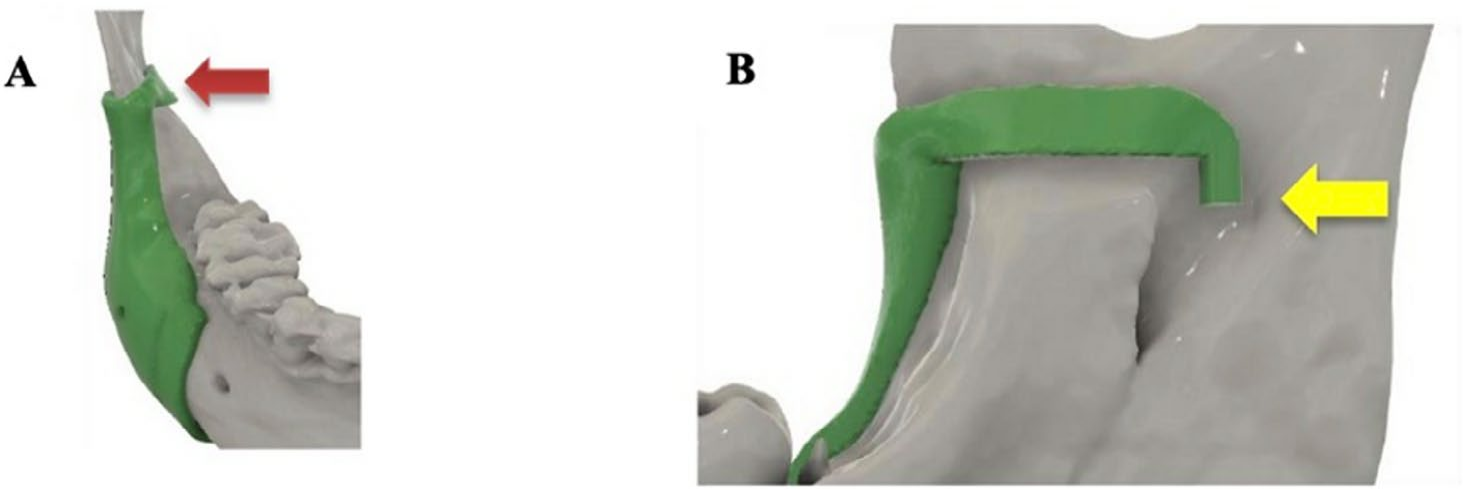
Key design features of the custom three‐dimensional (3D) surgical guide. (A) Lateral extension positioned along the anterior border of the mandibular ramus to assist guide orientation and stability during placement (red arrow). (B) Posterior lingual stop extending to the posterior border of the ramus to facilitate reproducible positioning of the guide (yellow arrow).

CT DICOM datasets of subjects' mandible were segmented in 3D Slicer [19], exported as a mesh, refined in Mesh‐mixer (Autodesk Inc., San Rafael, CA, USA) (Figure 2A), and saved as STL. Final CAD design was completed in Blender (Blender Foundation, Amsterdam, the Netherlands) to define guide thickness, osteotomy slots/trajectories, and fixation screw holes, then 3D‐printed in acrylic resin (FLSGAM01). All guides were 3‐mm thick and incorporated two lateral mini‐screw retention holes and a ramus insertion slot for additional stability (Figure 2B,C).

**FIGURE 2:**
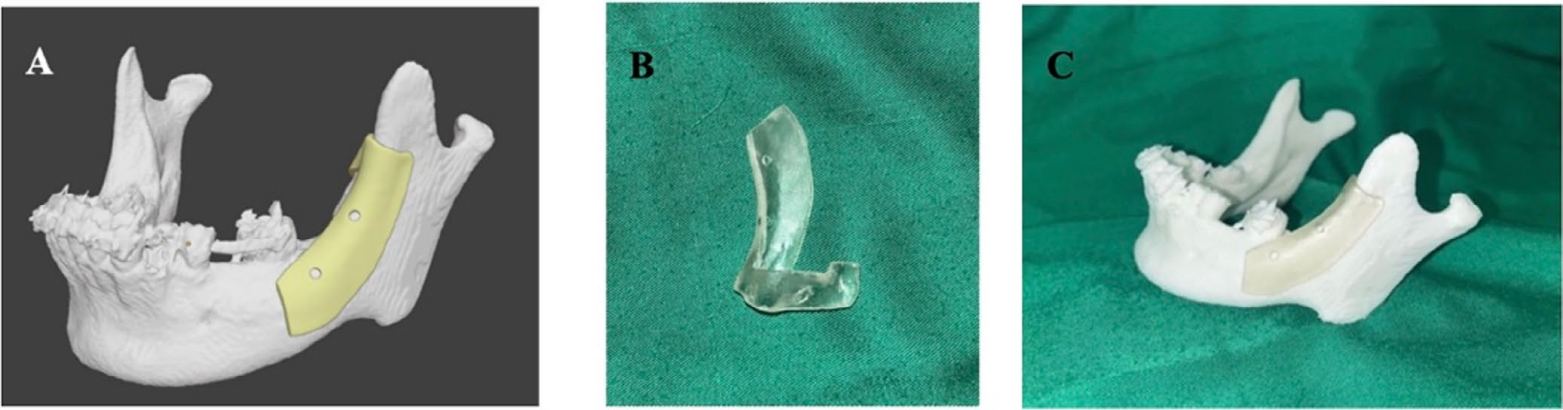
Design and fabrication of the custom three‐dimensional (3D) surgical guide. (A) Virtual design of the patient‐specific guide on the mandibular model. (B) Fabricated transparent resin surgical guide. (C) Verification of guide adaptation on the corresponding three‐dimensional printed mandibular model.

### 2.2 Surgical Procedure

The intervention was a custom 3D surgical cutting guide used during SSO based on the Obwegeser–Dal Pont–Hunsuck–Epker modification. The planned osteotomy included (1) a medial horizontal cut at the lingula extending anteriorly along the ramus slightly beyond the medial cortex, (2) a sagittal cut along the anterior ramus toward the first–second molar region, and (3) a lateral vertical buccal corticotomy from the inferior mandibular border to the sagittal cut level. In every case, one mandibular side was treated using the guide (Figure 3) while the other side was performed conventionally without guide in a single session by one experienced oral and maxillofacial surgeon (third author) [19]. The osteotomy was performed using a piezoelectric device, followed by osteotomes and a spreader to complete the split.

**FIGURE 3:**
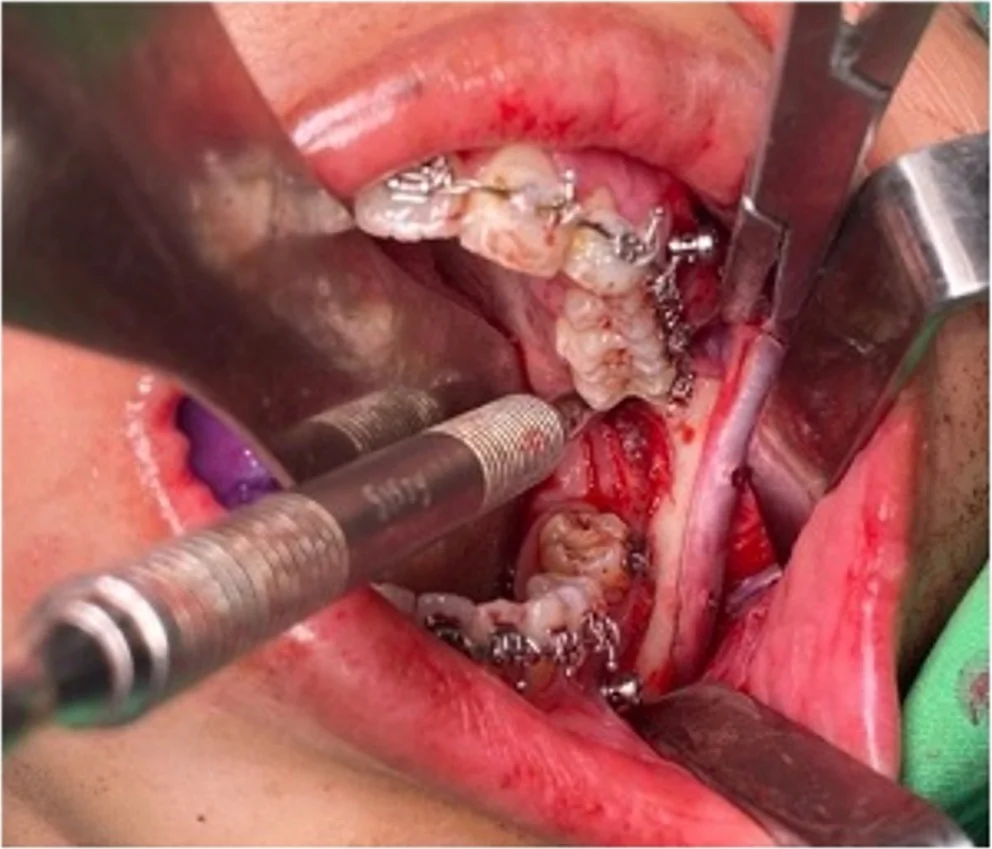
Intraoperative placement of the custom three‐dimensional (3D) surgical guide on the lateral surface of the mandibular ramus before performing the sagittal split osteotomy.

### 2.3 Outcomes and Postoperative Assessment

The primary outcome was postoperative LSS classified on CT‐based 3D mandibular evaluation according to Plooij et al. LSS 1 was described as a Hunsuck‐type vertical split toward the inferior mandibular border; LSS 2 was a horizontal split toward the posterior border of the ramus; LSS 3 traversed the mandibular canal and extended toward the inferior border; and LSS 4 included other configurations and was considered unfavourable [20].

Secondary outcomes were (1) the position of the lateral osteotomy end (LOE) at the inferior mandibular border, assessed using cross‐sectional CT images and 3D mandibular models, and (2) intraoperative inferior alveolar nerve exposure (NE), recorded during surgery. LOE was classified as lingual (L), inferior (I), or buccal (B) using the criteria of Song and Kim [21]. IAN exposure was categorized intraoperatively into three levels, NE1 (fully embedded, not visible), NE2 (partially exposed but confined within the distal segment), and NE3 (fully exposed with detachment from the distal segment), adapted from previous studies evaluating intraoperative inferior alveolar nerve exposure during sagittal split ramus osteotomy [22]. Intraoperative nerve exposure does not necessarily indicate nerve injury or long‐term neurosensory disturbance. There were no missing data for the primary or secondary outcomes.

Inter‐rater reliability was assessed for CT‐based categorical outcomes, including LSS and LOE, by two independent raters. Intraoperative nerve exposure was recorded during surgery and was not included in the CT‐based inter‐rater reliability analysis. In this study, assessments were performed by the first author (Rater 1) and an oral and maxillofacial radiology specialist (Rater 2), both of whom independently evaluated all outcome variables using predefined criteria. The evaluations were conducted simultaneously based on postoperative CBCT images.

### 2.4 Sample Size and Statistical Analysis

Given the absence of prior paired split‐mouth data for estimating the expected effect size of the primary outcome, a formal a priori power calculation was not feasible at the time of study design. Therefore, Federer's formula was used pragmatically to estimate the minimum sample size for this exploratory study [23]. Using (*t* − 1)(*n* − 1) ≥ 15 with two comparison conditions yielded a minimum of 16 mandibular sides, corresponding to at least 8 patients in a split‐mouth design. Recruitment was concluded after 15 patients had been enrolled, contributing 30 mandibular sides for paired analysis. No interim analyses or formal stopping rules were applied.

Statistical analysis was performed in IBM SPSS Statistics version 26 (IBM Corp, Armonk, NY, USA). Inter‐rater reliability between two raters was evaluated using Cohen's Kappa, with values interpreted as substantial (0.61–0.80) or almost perfect (0.81–1.00) agreement. Given the paired split‐mouth study design and the categorical nature of the outcome variables, statistical comparisons were performed using the Wilcoxon signed‐rank test for LSS and NE, while the Stuart‐Maxwell marginal homogeneity test was applied for LOE analysis, with *p* < 0.05 considered significant. No subgroup, sensitivity or post hoc analyses were performed.

## 3 Results

Fifteen individuals were screened and qualified for participation (Table 1). All analyses were performed at the mandibular side level, with each patient contributing paired observations (guided and contralateral non‐guided side). All participants received identical perioperative care for both the guided and non‐guided sides. Postoperative assessment was performed using CT imaging at 2 weeks. To ensure consistency of outcome evaluation, inter‐rater reliability was assessed using Cohen's Kappa coefficient. This showed excellent agreement, with kappa values ranging from 0.81 to 1.00. The level of agreement between the first author (Rater 1) and the oral and maxillofacial radiology specialist (Rater 2) was very high, with Cohen's kappa values of 0.851 for LSS and 0.818 for LOE.

**TABLE 1:** Characteristics of study subjects based on age, sex, skeletal pattern, and mandibular movement.

Case	Age (years)	Sex	Skeletal pattern	Mandibular movement
A	25	Male	III	Setback
B	21	Female	II	Advancement
C	26	Male	III	Setback
D	33	Male	III	Setback
E	26	Male	III	Setback
F	26	Female	III	Setback
G	24	Female	III	Setback
H	28	Male	III	Setback
I	23	Female	II	Advancement
J	22	Male	III	Setback
K	36	Male	III	Setback
L	28	Female	III	Setback
M	29	Male	III	Setback
N	34	Female	III	Setback
O	27	Male	III	Setback

A total of 15 patients were included in the study, of whom 9 (60%) were male and 6 (40%) were female. The patients' ages ranged from 21 to 36 years. The mean age of female patients was 26 years, while the mean age of male patients was 28 years. The majority of patients exhibited skeletal Class III deformity, accounting for 13 patients (86.7%), while a skeletal Class II pattern was identified in 2 patients (13.3%). All procedures were performed by a single surgeon (third author) over the study period from 2022 to 2025. The surgeries were carried out at two centres, with 9 procedures conducted at Universiti Malaya Medical Centre and 6 at University of Indonesia Hospital.

### 3.1 Lingual Split Scale

Most mandibular sides in the guided group demonstrated favourable lingual split patterns. Paired analysis using the Wilcoxon signed‐rank test showed a statistically significant difference in lingual split scale distribution between guided and non‐guided sides (*Z* = −2.041, *p* = 0.041). LSS 1 was more frequently observed on guided sides (86.7%) compared with non‐guided sides (46.7%), whereas LSS 2 and LSS 3 patterns were more commonly identified on non‐guided sides (26.7% each) than on guided sides (6.7% each). No LSS 4 pattern was observed in either group.

### 3.2 Nerve Exposure

Paired analysis using the Wilcoxon signed‐rank test revealed no statistically significant difference in nerve exposure grade between guided and non‐guided sides (*Z* = −0.302, *p* = 0.763). NE 1 was observed in 80.0% of guided sides and 73.3% of non‐guided sides. NE 2 occurred more frequently on non‐guided sides (26.7%) than on guided sides (13.3%), while NE 3 was identified on one guided side (6.7%) and was not observed on non‐guided sides.

### 3.3 Lateral Osteotomy End

Based on the data presented in Table 2, inferior lateral osteotomy end (LOE) patterns were more frequently observed on guided sides (73.3%) compared with non‐guided sides (33.3%), whereas lingual LOE patterns occurred more frequently on non‐guided sides (40.0%) than on guided sides (13.3%). Paired analysis using the Stuart–Maxwell test of marginal homogeneity demonstrated a statistically significant difference in LOE distribution between guided and non‐guided sides (Standard MH Statistics = −2.043, *p* = 0.041).

**TABLE 2:** Comparative analysis of lingual split scale, nerve exposure, and lateral osteotomy end between guided and non‐guided sides in patients undergoing SSO.

Outcomes	Guide side (*n* = 15)	95% CI	Non‐guided side (*n* = 15)	95% CI	Statistical test	*p*
Lingual split scale (LSS)						
LSS 1	13 (86.7%)	0.62–0.96	7 (46.7%)	0.25–0.70	Wilcoxon signed‐rank test *Z* = −2.041	0.041*
LSS 2	1 (6.7%)	0.01–0.31	4 (26.7%)	0.11–0.52		
LSS 3	1 (6.7%)	0.01–0.31	4 (26.7%)	0.11–0.52		
LSS 4	0 (0.0%)	0.00–0.21	0 (0.0%)	0.00–0.21		
Nerve exposure (NE)						
NE 1	12 (80%)	0.55–0.93	11 (73.3%)	0.48–0.89	Wilcoxon signed‐rank test *Z* = −0.302	0.763
NE 2	2 (13.3%)	0.04–0.38	4 (26.7%)	0.11–0.52		
NE 3	1 (6.7%)	0.01–0.31	0 (0.0%)	0.00–0.21		
Lateral osteotomy end (LOE)						
Inferior	11 (73.3%)	0.48–0.89	5 (33.3%)	0.15–0.58	Stuart–Maxwell test of marginal homogeneity Standard MH statistic = −2.043	0.041*
Lingual	2 (13.3%)	0.04–0.38	6 (40.0%)	0.19–0.64		
Buccal	2 (13.3%)	0.04–0.38	4 (26.7%)	0.11–0.52		

*Note:* Wilcoxon signed‐rank test was used for paired ordinal outcomes (LSS and NE). Stuart–Maxwell test of marginal homogeneity was used for the paired nominal outcome (LOE).

Abbreviation: CI, confidence interval.

* *p* < 0.05 is considered statistically significant.

Figure 4 presents representative 3D mandibular reconstructions and cross‐sectional images comparing sagittal split osteotomies performed with and without a custom 3D surgical guide. The lingual split scale was classified according to the modified system proposed by Plooij [20], showing a more consistent fracture trajectory on the guided side compared with the unguided side. Evaluation of the lateral osteotomy end, assessed based on the classification described by Song and Kim [21], showed a predominantly inferior termination on the side treated with the surgical guide, whereas greater variability was observed on the side without guidance.

**FIGURE 4:**
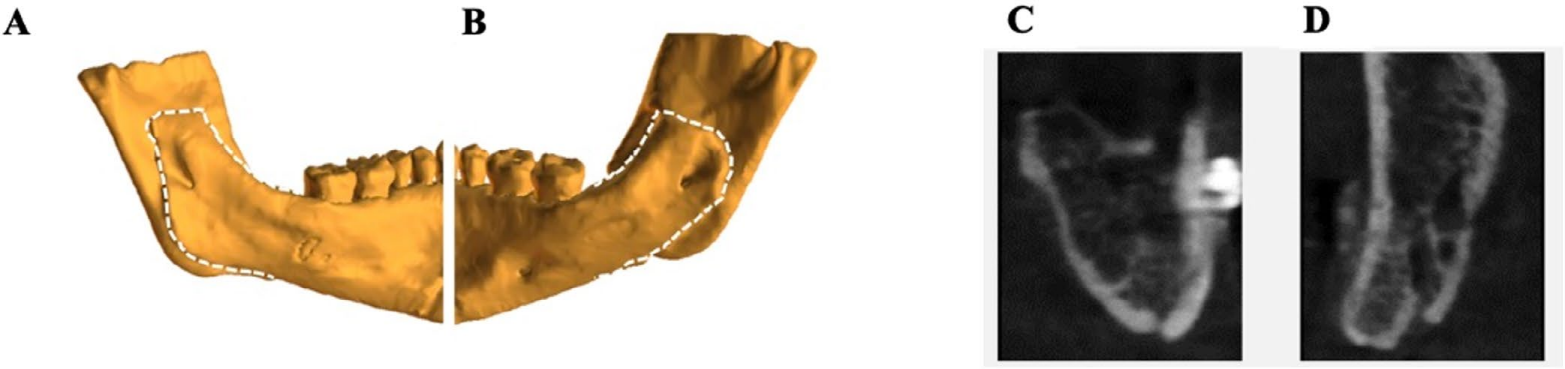
Representative postoperative three‐dimensional mandibular reconstructions and cross‐sectional CT images comparing guided and non‐guided sagittal split osteotomies. (A) Three‐dimensional reconstruction of the guided side. (B) Three‐dimensional reconstruction of the contralateral non‐guided side. (C) Cross‐sectional CT image of the guided side. (D) Cross‐sectional CT image of the contralateral non‐guided side.

## 4 Discussion

Sagittal split osteotomy (SSO) is associated with various reported complications. Despite advancements in technique and instruments, surgeons continue to encounter technical challenges and are confronted with various intraoperative and postoperative complications [24]. Overall, this surgical procedure is considered safe. However, there are a few complications which are commonly encountered in SSO, such as unfavourable fractures during osteotomy, infection postoperatively, and neurosensory disturbance [25]. In a meta‐analysis done by Verweij et al., they noted a mean pooled incidence of 2.3% per SSO [6].

Surgical cutting guides could be fabricated with two different methods, manual or digital. Both methods require pre‐operative CBCT images to generate a 3D digital mandible model. Digitally designed and 3D‐printed guides may improve the reproducibility of osteotomy transfer compared with manually fabricated guides, because the final guide is generated directly from the virtual surgical plan. The advancement of 3D printing technology offers a potential solution to these issues. With 3D printing, the surgical guide is produced as a reverse‐engineered product of the surgical design [26]. In this study, surgical guides were all fabricated digitally to improve dimensional consistency and reduce fabrication‐related distortion. The guides were manufactured using in‐house 3D printing with compatible resin and post‐processed.

This exploratory split‐mouth randomized clinical trial suggests that custom 3D‐printed surgical guides may improve osteotomy control during SSO, particularly for parameters reflecting osteotomy trajectory and split behaviour, such as LSS and LOE. Although guided osteotomies were associated with improved osteotomy and procedural control, no statistically significant difference in intraoperative nerve exposure was observed between guided and non‐guided sides. Therefore, the present findings should not be interpreted as evidence that the guide reduces nerve exposure or neurosensory risk [27, 28]. This is consistent with 3D observations that irregular lingual split deviations often originate from variability in the medial osteotomy trajectory. By stabilizing and constraining the planned cutting path, a guide can reduce such deviations and promote a more reproducible split geometry [20]. In the past decade, piezoelectric devices have gained popularity in orthognathic surgery due to their lower invasiveness. Piezoelectric technology employs vibrations at a frequency ranging from 24 to 29 kHz. The resultant micromovements or vibrations are adept at selectively targeting the mineralized tissues, particularly bone [29]. Accordingly, osteotomy was performed using a piezoelectric device, followed by osteotomes and a spreader.

The clinical relevance of improved split predictability lies in the distribution of split patterns. LSS 1 is generally considered the most desirable configuration because it provides broad bony contact between proximal and distal segments, which supports stability and bone healing. In contrast, unfavourable patterns, particularly LSS 4, are associated with a higher risk of bad split and may involve the region of the inferior alveolar canal. Interestingly, Feng Li et al. noted that LSS 2 tends to occur more frequently in younger patients due to the higher elasticity of their cortical bone, which allows external forces to be transmitted to the posterior spongiosa, resulting in this particular split pattern [30].

In the present study, guided osteotomies were associated with a more controlled and favourable split pattern profile, indicating that standardizing the osteotomy pathway may help shift outcomes toward configurations with more reliable segment contact. These findings align with earlier 3D work showing that extending the medial osteotomy posteriorly beyond the mandibular foramen increases the likelihood of favourable LSS 1 and reduces less favourable patterns [31].

Anatomical risk factors also help contextualize the potential value of guide assistance. In a large‐scale study, Jiang et al. reported a bad split incidence of 4.15% [11]. While a guide cannot alter anatomy, it can improve adherence to a planned ‘safe corridor’ and reduce operator‐dependent deviations that may bring the osteotomy closer to the canal, particularly in high‐risk morphologies [32]. Collectively, these observations support the role of custom guides in mitigating technical sources of variability, an important contributor to unpredictable osteotomy outcomes [33].

Regarding nerve outcomes, no statistically significant difference in NE was observed between guided and unguided sides. However, a higher proportion of NE1 occurred in the guide group, although the difference was not statistically significant. This is consistent with Kaba et al., who reported no significant association between lingual split scale and postoperative neurosensory disturbances (NSD) [34]. The absence of a significant group difference is biologically plausible because nerve exposure and NSD are multifactorial: beyond fracture configuration, they depend on surgical handling, individual anatomy, cortical thickness, and intraoperative force transmission during splitting [35]. Given that reported IAN injury rates after sagittal split osteotomy can be substantial (40%–60%) [36], any technique that may improve control of osteotomy trajectory and reduce unintended deviations toward the canal may still be clinically meaningful, even if short‐term exposure differences are modest [37].

In contrast to Al‐Ahmad et al., the present study did not demonstrate a significant difference in nerve exposure between guided and non‐guided side [38]. Although one NE3 case was observed on the guided side, the overall distribution of NE grades did not differ significantly between groups. Direct comparison between studies is limited by differences in surgical technique, nerve exposure classification, and sample size. Cutting guides have been described in orthognathic surgery, where the osteotomy trajectory and anatomic position can be adjusted via virtual surgical planning preoperatively, which may help reduce the risk of damaging vital structures during surgery [39].

The LOE analysis provides additional insight into how guide assistance may influence split behaviour. LOE is clinically important because the direction and endpoint of the lateral corticotomy influence the split pattern, segment stability, and risk of unfavourable fractures [30]. In this study, the guided sides showed a significantly different LOE distribution, most often terminating at the inferior border. This finding aligns with the mechanism proposed by Feng Li, who reported that deeper or more lingually positioned LOE increases the likelihood of LSS 3, whereas inferior termination supports more favourable split patterns [30]. It is also consistent with Song and Kim, who identified lateral corticotomy direction and endpoint as key contributors to unfavourable splits [21]. In practical terms, the guide likely acted not only as a cutting template but also as a directional stabilizer that improved reproducibility of the lateral cut endpoint, supporting wider and more symmetric bony contact between segments.

This study has several strengths. The split‐mouth randomized design reduces inter‐individual confounding and enables within‐patient comparison of guided versus unguided osteotomies, with each participant serving as their own control. Outcome assessment was performed on postoperative CT imaging with independent dual rating by an expert, and inter‐rater agreement was excellent (Cohen's kappa 0.81 to 1.00), supporting the internal validity of the measurements.

Several limitations should be acknowledged. First, the sample size was determined pragmatically without a formal paired‐design power calculation, which may have limited the ability to detect small but clinically relevant differences, particularly for nerve exposure. Given the exploratory nature and limited sample size, more complex paired statistical modelling was not considered feasible, and future studies should use paired‐design sample size estimation. Second, the study population consisted predominantly of skeletal Class III patients (86.7%), and only 2 patients (13.3%) presented with Class II patterns. Meaningful subgroup analysis between Class II and Class III patients was therefore not feasible, and caution is warranted when extrapolating these findings to other dentofacial deformity patterns. Third, the study assessed short‐term anatomical outcomes and intraoperative nerve exposure only. The findings cannot be extrapolated to long‐term neurosensory recovery, skeletal stability or bone remodelling. Fourth, although the guide may improve the planned osteotomy trajectory, it does not directly control osteotomy depth or the final fracture propagation during splitting. Unfavourable split patterns may still be influenced by cortical thickness, canal‐to‐buccal cortex distance, ramus morphology, splitting technique, and surgeon experience. Future studies with larger cohorts, longer follow‐up, patient‐reported neurosensory outcomes, and anatomy‐informed risk stratification are needed to determine which patients benefit most from guide‐assisted SSO.

## 5 Conclusion

In this exploratory split‐mouth randomized controlled trial, the use of a custom 3D‐printed surgical guide was associated with a more favourable distribution of lingual split patterns and lateral osteotomy end positions compared with conventional freehand osteotomy. No significant difference in intraoperative inferior alveolar nerve exposure was demonstrated between guided and non‐guided sides. Because the study was exploratory, used a small sample, and assessed only short‐term anatomical outcomes, these findings should be interpreted as hypothesis‐generating. Given the predominance of skeletal Class III patients in the study population, they are primarily applicable to this patient group. Adequately powered randomized trials with longer follow‐up and clinical neurosensory endpoints are needed to determine whether guide‐assisted osteotomy translates into improved patient‐relevant outcomes.

## Funding

This study was funded by the Directorate of Funding and Research Ecosystem, Universitas Indonesia, through the Publikasi Terindeks Internasional (PUTI) Q1 Grant (Grant No. PENG‐003/UN2.RST/PPM.00.00/2025).

## Ethics Statement

The studies involving human participants were reviewed and approved by the Ethics Committee of University of Indonesia Hospital (S‐159/KETLIT/RSUI/VII/2025). The Faculty of Dentistry Ethics Committee at Universiti Malaya accepted the research protocol, which was given the reference number DF OS2211/0024 (P). This research was also approved by the ethical committee at the Faculty of Dentistry, University of Indonesia (Reference Number: 78/Ethical Approval/FKGUI/V/2025). The trial was registered in the International Randomized Controlled Trial Number (ISRCTN) with trial number ISRCTN35139147.

## Conflicts of Interest

The authors declare no conflicts of interest.

## Supporting information


**Data S1:** Consort flow diagram.

## Data Availability

The data that support the findings of this study are available on request from the corresponding author. The data are not publicly available due to privacy or ethical restrictions.
