# Supplementary material for: Evaluation of a Custom Three‐Dimensional Surgical Guide in Mandibular Sagittal Split Osteotomy: An Exploratory Split‐Mouth Randomized Controlled Trial
Source: Orthod Craniofac Res. 2026 Jul 16;29(6):1144–52. doi: 10.1111/ocr.70163 (PMC13752649; doi:10.1111/ocr.70163)
Supplement: Supplementary file 1 [file OCR-29-1144-s001.docx]

Supplementary materials

S1. Consort flow diagram
